# Comparative Modeling of Protein Structure—Progress and Prospects

**DOI:** 10.6028/jres.094.010

**Published:** 1989

**Authors:** John Moult

**Affiliations:** Center for Advanced Research in Biotechnology University of Maryland 9600 Gudelsky Drive Rockville, MD 20850

**Keywords:** comparative modeling, electrostatics, hydrophobicity, protein structure, sequence alignment

## Abstract

Comparative modeling of protein structure is a process which determines the three-dimensional structure of protein molecules on the basis of amino acid sequence similarity to experimentally linown structures. The procedure is facilitated by the growing database of protein structures obtained from crystallography. In this review a series of stages in the modeling process are identified and discussed. These are: (i) obtaining a reliable amino acid sequence of the structure of interest, (ii) producing a structurally correct sequence alignment, (iii) identifying which structural features are conserved between target and parent structures, (iv) modeling the new pieces of structure, and (v) tests of reliability.

## Introduction

Soluble, globular protein molecules are now some of the best understood components of biological systems. X-ray structures of several hundred structures [1], together with extensive biochemical studies, have led to detailed models of the mechanism of action of these molecules, particularly enzymes. Ever since it was established that in many cases the amino acid sequence is sufficient to determine the three-dimensional structure of such molecules, without the aid of additional biochemical machinery [2], the task of predicting structure from sequence has received a great deal of attention. However, a solution to the general problem still eludes us. Meanwhile, an accumulation of structures which have been determined by crystallography, together with the realization that most proteins are closely related in sequence to a number of others [3], has opened up the possibility of using database approaches to structure determination. These methods, utilizing the set of known structures, are known as comparative protein modeling, or sometimes homologous modeling. As we shall see, the techniques employed use database information in a very indirect manner, with emphasis usually on numerical algorithms rather than the small amounts of data these draw on.

## Usefulness

Comparative modeling is a worthwhile activity in two ways: it tests and extends our knowledge of the principles that determine protein structure, and it allows functional insight to be obtained from sequence data quickly, instead of waiting for the laborious process of protein production, purification, and crystallography. With DNA sequencing technology producing sequences at a very high rate it is likely that many, many proteins will be investigated by modeling rather than by structure determination. As an example of this, consider the case of the T-cell defense mechanism of the immune system. When a cytotoxic T-cell destroys a foreign cell, a number of genes are activated. Detection of these at the messenger RNA level has led to sequence data. Sequence comparison shows these genes to express serine proteases, facilitating comparative modeling of their three-dimensional structure. The models allow deductions to be made about the specificity of these enzymes [4], Thus, the picture of the T-cell defense mechanism is enhanced, based on modeling alone.

## How Difficult Is It?

The degree of difficulty involved in producing a model of a protein by the methods of comparative modeling depends on two main considerations: How accurate and reliable does the model need to be, and how homologous is its sequence with that of the most closely related known structures? Comparison of how similar related structures are [5], has shown that there is an exponential divergence of structure with decreasing sequence similarity. Fifty percent or more identity of sequence ensures a model for which most regions are accurate to approximately 1 Å at the α-carbon positions. With less than 30% identity of sequence few details of a model can be relied upon. These overall figures are often irrelevant, however. If, for instance, the model is needed for the design of molecules that will bind tightly at some site, then only a local subset of the structure is important. An example of such ligand binding oriented modeling is the popular exercise of modeling of the enzyme renin with a view to designing inhibitors which will act as anti-hypertensives [6], In such cases, it matters little if 95% of the model is accurate, if the 5% that is wrong involves the substrate binding site. Further, at least a 1 Å root mean square (rms) accuracy is needed in the relevant regions, for all atoms likely to interact with a ligand. One should be cautious, then, of claims that a model is mostly correct. Modeling the highly homologous regions is often trivial, and their correctness may be as irrelevant as in the case of the curate’s egg, that was only rotten in parts.

## How to Proceed

Progress in this area has been fairly slow, mainly because of a lack of feedback on the accuracy of the models produced. The situation is now changing, as more previously modeled structures become known through crystallography. In this review, I shall draw on a comparison between modeled and experimentally determined versions of the same protein. The case of the relationship between a trypsin-like molecule from the bacterium Streptomyces gresius (SGT) and bovine trypsin (BT) will serve to illustrate many of the pitfalls that litter the path of model building. SGT is 33% identical in sequence with BT, on a structurally based alignment, and so represents a moderately difficult modeling problem. The structure of SGT was modeled twice [7,8] before the x-ray structure was determined. Read et al. made a careful comparison of the modeling results with the x-ray structure [9]. Armed with such insight, one may identify a set of stages in producing a model, and consider for each of these how reliable current techniques are, and what are the prospects for improving them.

This review is intended as a practical guide to comparative modeling—what to worry about, how to do things, and when to believe your own and other people’s claims of success.

### Stage 1: Obtaining a Reliable Amino Acid Sequence

If there are errors in the amino acid sequence of the protein to be modeled there must be errors in the resulting structure. Problems in this area can reverberate through the subsequent stages, aggravating already difficult steps. It is common experience for crystallographers to find discrepancies between the amino acid sequence reported in the literature and that indicated by electron density maps. Sometimes these are due to isoforms of the protein, but often they turn out to be sequencing errors. In the case of SGT, the omission of two residues from the amino acid sequence [10] happened to occur in one of the most difficult to align regions, and was one of the reasons why all proposed alignments were wrong (in a structural sense—see below) in that stretch of chain. Useful checks that the modeler should make in this area are to look at the sequences of any related proteins that are available, to compare DNA and amino acid level sequences if possible, and to consult the original sequence literature, if obtainable.

### Stage 2: Producing a Structurally-Based Sequence Alignment

In order for modeling to begin, the sequence of the target structure must be aligned with those of the relevant known ones. The alignment required is not necessarily that which produces the greatest number of identities of residue type between the sequences. Rather, it is the one which correctly assigns structural roles to the residues of the target structure. Comparison of alignments based on sequence with those based on structure shows that once the degree of sequence identity falls below approximately 40%, errors are inevitable. In the case of SGT, sequence alignments with BT range between 78 and 91% accuracy [9] for the structurally equivalent residues. The primary problem here is where to put insertions and deletions in the target structure relative to the known ones.

An important point to bear in mind is that more than one parent structure may be useful as the basis for modeling. For instance, in the case of the Hannuka factor T-cell protease, parts of the molecule are most similar in sequence to rat mast cell protease, while others are closer to bovine trypsin [4]. SGT is generally closest in sequence to BT, but one loop of five residues is similar to an equivalent region which occurs only in chymotrypsin. Greer observed this, and was thus able to correctly determine the structure of that piece [8]. That author has referred to this approach as the “spare parts” approach to comparative modeling.

Techniques are beginning to emerge which hold promise of producing structurally correct alignments. One approach makes use of the observation that insertions and deletions are unlikely to occur in regions of secondary structure, such as helices and sheets, so that a large penalty function can be used in such regions in making the alignment [11].

Consideration of the underlying reason for the structural equivalence of residues leads to a more general approach to this step: An extraordinary property of functional proteins is that each amino acid is immobilized relative to the structure as a whole by interactions with the surrounding residues. In this sense, as Havel has pointed out, they are tensegrity structures. This property distinguishes them from other heterogeneous polymers, and presumably from almost all possible random protein sequences. Thus, any pair of structures with related sequences will to a large degree maintain these stabilizing interactions for each residue, and where this is not the case, new ones must be substituted. Consideration of the conservation of interactions in going from one structure to another provides a potentially powerful method of checking and modifying a sequence alignment.

As an example of how this might work, consider the most problematic region of alignment between SGT and BT:

**Figure:**
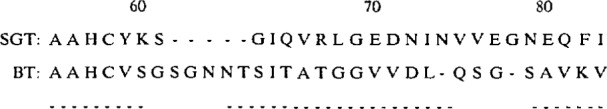


The correct alignment is shown here, with the chymotrypsin residue numbering convention. The bars beneath the sequences indicate residues which are structurally equivalent in the two structures (based on data from [9]). Residues G77 and S79 of SGT are the two not reported in the amino acid sequence. The structures of the central portion of this stretch are quite similar, but there are only two residue identities: 163 and G69. There are alternative alignments possible which also have two identities, always involving alignment of V70 in SGT with V75 of BT. An effective alignment algorithm must therefore be able to choose the correct one of these alternatives. Examination of the BT environment of the residues involved shows how this is possible: 163 is involved in extensive hydrophobic core interactions with residues from three remote parts of the structure, and any alignment which does not position an appropriate hydrophobic residue at this position is clearly not viable. G69 is in a buried tight turn, and the surrounding pieces of chain leave no room for any substantial side chain at this position. Less definitively, it is also in the left-handed alpha helix conformation, reducing the probability that any other residue could be accommodated. In contrast to these severe restrictions, V75 makes no hydrophobic contacts, and a number of different side chains can easily be used instead.

In a full implementation of this method, the contacts of every residue in the target structure will be compared with the contacts for the structurally equivalent residue in the parent structure. Contacts are listed by type: nonpolar to nonpolar, polar to polar, charge to charge, and combinations of these. A scoring scheme is used to assess list similarity. Alternative positions for the insertion or deletion of residues may then be assessed on the basis of the conservation of interaction scores. This approach also has application in the next step in the modeling process.

### Stage 3: Identifying Conserved Structural Features

Once a satisfactory sequence alignment has been obtained, a preliminary model can be produced by simply substituting each changed amino acid residue in the parent structure(s) for the appropriate one in the target structure. An assessment must now be made of which residues have the same relationship to the rest of the structure as in the parent molecule, and which different ones. Traditionally, this step has been done by inspection: An obvious place to begin is with the places where there is good sequence homology between parent and target structure. One would normally assume that such regions will have closely similar structures. Although this is generally true, there are exceptions. In SGT, for example, there is one region of apparent reasonable sequence homology (146–152 [9]) where main chain α-carbon positions differ by more than 1.9 Å compared with the parent BT structure:

**Figure:**
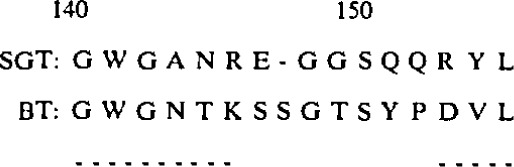


The method of environment characterization outlined above can be used to appreciate why there is a breakdown in structural similarity in this stretch: G148 is held in position in BT by polar interactions with the side chains of N143, S147, and T149. In SGT, these residues are all atrophied, and nonpolar, becoming A, A, and G, respectively. Interactions of the side chain of SI50 in BT are weak, so that it largely relies on the surrounding residues to hold it in position, and these are not conserved. Application of the environment classification algorithm would thus appear to be able to identify which features of structure are conserved in the target molecule.

### Stage 4: Building New Structural Regions

At the end of the previous step, a list of regions which need remodeling, ranging from single residue side chains to whole stretches of chain, has been produced. These regions must now be constructed in some manner. A number of approaches are possible:

- Human judgment. The most usual method. An operator sitting in front of a graphics system inspects the region to be modeled, and draws on his experience to suggest one or more possible structures. In simple cases, such as positioning a single side chain, this may be effective. In general, though, there are too many possibilities to be considered, and human judgment tends to be more like human prejudice than a rational consideration of the options. In the modeling of SGT, Read et al. [9] found that all attempts to construct segments of three or more residues led to serious errors. There is also a problem of nonreproducibility inherent in such a subjective procedure.
- Databases of known structures. Since we are interested in quite short lengths of polypeptide chain, it seems likely that useful information can be obtained directly from the database of known structures. For lengths of chain up to five residues long it is indeed true that the set of known structures will usually contain one or more examples with an rms on α-carbon atoms of 1.0 Å or better to any target structure. The issue becomes one of selection. Sequence homology for such short stretches is not an indication of similarity of conformation, even in the limit of identical sequences [12]. Knowing the positions of the ends of the stretch turns out to be a powerful constraint on possible conformations. For up to five residues, this information may be used to select a small set—typically 5 to 20 conformations, separated from each other by 1 Å rms or more in α-carbon rms space. This is particularly useful for selecting a conformation that best fits a poor quality electron density map [13]. However, when no experimental information is available to limit the choice, the outcome is a set of possible α-carbon traces, and no means of choosing between them. Further, the database is only large enough to provide a description at the α-carbon level—once all atoms of the backbone and side chains are added the number of possible conformations increases dramatically. Such a detailed level of description is needed both to provide a useful model of the structure, and to enable energetic criteria to be used to distinguish between possibilities. Because the number of possible all atom structures is so great, the database of structures will never be large enough to contain all five residue stretch conformations at the 1 Å accuracy level. Still, this method has the merit of speed, and may be used to guide operator thinking to extend the scope of method (a). The graphics program **FRODO** elegantly incorporates the required database, and other programs will soon do so as well.
- Molecular dynamics simulation. The most straightforward of a set of approaches which use energetic criteria in some form to select a conformation. The idea here is that an arbitrary conformation of the region to be modeled is selected, and then a molecular dynamics trajectory of the local region of structure is performed, sufficiently long that the correct conformation will be encountered, and may be recognized by its low energy compared with the alternatives. Contemporary empirical potentials do seem to be able to represent structure at a level approaching the 1 Å rms level [14], so that from a discrimination standpoint this approach is viable. There are serious difficulties with the length of the simulation required, however. Getting from an arbitrary starting conformation to the correct one may entail rearrangements of the local structure which can only be achieved by unfolding a large part of the protein, so that no affordable simulation will be able to reach the correct structure. Possible approaches to overcoming this obstacle are to use an elevated temperature, making the surmounting of conformational energy barriers more frequent; a lowering of the van der Waals repulsive energies so that pieces of chain may pass through each other; and using a number of starting conformations, in the hope that one of them will be within the convergence range of the method. So far, these approaches have not been demonstrated to be effective.
- Distance geometry. If sufficient interactions can be identified, these may be used as restraints to restrict the number of possible conformations, in much the same manner as NOE distances are used to define peptide and protein structure with this method [15]. Possible restraints are connection to the rest of the structure, and liganding to a bound group, such as an ion [16]. It may also be feasible to investigate combinations of possible interactions, such as salt bridges or hydrophobic contacts. Such restrictions may provide a powerful approach to this type of modeling problem, but their potential has yet to be explored.
- Systematic Conformational Search (SCS). This method consists of two steps: Generate all possible conformations, and hen use some sort of discriminatory functions to choose one close to the correct structure. Several groups [14,17–19] are pursuing this approach, with somewhat different methodologies. Here I will summarize our own work [14].

“All possible conformations” implies sampling the conformational space sufficiently finely that at least one conformation will be generated within the desired deviation from the correct structure. The limit of acceptable deviation is dictated by the ability of the discriminatory functions to identify a structure as nearly correct, and to distinguish which of two possible conformations is actually more correct. In practice, this means sampling the conformational space with a density of about 1 Å rms.

For the discriminatory functions to be effective, an all atom description of the structures is at present required. There are astronomical numbers of such possible structures at the 1 Å rms density level, even for short pieces of chain—of the order of 10^4^*^n^*, where *n* is the number of residues. Thus the approach is impractical, unless ways can be found of reducing the number of conformations that need to be considered. This can be done by using rules that protein structures obey as filters to reduce the number of conformations. Useful rules are simple: For example, the preference for ranges of dihedral angles, the avoidance of van der Waals clashes, and the restriction of joining the edges of the segments to the rest of the structure. In practice, however, none of these rules is completely obeyed: For instance, the energetic cost of van der Waals clashes is so high that they never occur in real structures. However, when structures are generated by finite sampling, very significant clashes will likely be present in the best structure generated. Such clashes must therefore be allowed for in deciding whether to accept the structure for further consideration. Nevertheless, for lengths of up to seven residues, depending on the sequence, the number of conformations can be reduced to a few thousand, and these may then be evaluated using the discriminatory functions.

Suitable discriminatory functions are the electrostatic energy of each conformation, and the amount of exposed hydrophobic area. Tests have shown that it is possible to first determine the conformation of electrostatically sensitive parts of a structure (main chain, polar and charged side chains) and then to consider the best conformation of hydrophobic side chains. Electrostatic energy is evaluated using an empirical force field [20], together with a solvent reaction field [21]. Exposed hydrophobic area is calculated using a standard surface area algorithm [22], and considering those atoms in the force field which carry zero partial charge to be hydrophobic.

These discriminatory functions are effective. Although they do not always select the very best structure generated, they do find one close to the best, and within approximately 1 Å rms of the correct (x-ray) structure [14]. Provided the previous stages have been successfully carried out, good structural models are produced. Further developments of the method are in progress, and hold promise of extending the number of residues which can be considered.

### Stage 5: Evaluation of Reliability

A central difference between modeling and experiment is that in the latter regime it is usually possible to test a conclusion against the data and know how reliable that conclusion is. This is not generally possible with a model. However, there are a number of ways of assessing the reliability of a protein model:

- (a) Note any uncertainties in the data for the original sequence, such as differences between amino acid and nucleic acid results, and substitutions compared with related proteins that appear unlikely. Also note uncertain or alternative possible sequence alignments with the parent structure(s).
- (b) Evaluate the exposed surface area of different types of residues in the resulting structure, and note any exposed hydrophobes, or buried charges. Although both situations do occur [23,24] they are sufficiently unusual to be worthy of suspicion.
- (c) Evaluate the packing of each group in the protein, and note any poorly packed regions, or cavities. Poor packing and cavities are present in proteins [25], but again, their presence in a model serves to focus attention on a particular suspect region.
- (d) Evaluate the electrostatic environment of all polar and charged groups. Note any overall unfavorable ones. Examination of refined x-ray structures shows that these are unusual in well ordered regions.

None of these criteria will yield a completely definitive answer. However, they will allow the probability of correctness of any feature in the structure to be assessed. The usefulness of these criteria has recently been reviewed [26]. This information can then be used in deciding whether to accept conclusions concerning function drawn from the model.
